# Nitric oxide: A core signaling molecule under elevated GHGs (CO_2_, CH_4_, N_2_O, O_3_)-mediated abiotic stress in plants

**DOI:** 10.3389/fpls.2022.994149

**Published:** 2022-11-01

**Authors:** Nkulu Rolly Kabange, Bong-Gyu Mun, So-Myeong Lee, Youngho Kwon, Dasol Lee, Geun-Mo Lee, Byung-Wook Yun, Jong-Hee Lee

**Affiliations:** ^1^ Department of Southern Area Crop Science, National Institute of Crop Science Rural Development Administration (RDA), Miryang, South Korea; ^2^ Laboratory of Molecular Pathology and Plant Functional Genomics, Kyungpook National University, Daegu, South Korea

**Keywords:** nitric oxide, greenhouse gases, stress signaling, nitrogen metabolism, abiotic stress

## Abstract

Nitric oxide (NO), an ancient molecule with multiple roles in plants, has gained momentum and continues to govern plant biosciences-related research. NO, known to be involved in diverse physiological and biological processes, is a central molecule mediating cellular redox homeostasis under abiotic and biotic stresses. NO signaling interacts with various signaling networks to govern the adaptive response mechanism towards stress tolerance. Although diverging views question the role of plants in the current greenhouse gases (GHGs) budget, it is widely accepted that plants contribute, in one way or another, to the release of GHGs (carbon dioxide (CO_2_), methane (CH_4_), nitrous oxide (N_2_O) and ozone (O_3_)) to the atmosphere, with CH_4_ and N_2_O being the most abundant, and occur simultaneously. Studies support that elevated concentrations of GHGs trigger similar signaling pathways to that observed in commonly studied abiotic stresses. In the process, NO plays a forefront role, in which the nitrogen metabolism is tightly related. Regardless of their beneficial roles in plants at a certain level of accumulation, high concentrations of CO_2_, CH_4_, and N_2_O-mediating stress in plants exacerbate the production of reactive oxygen (ROS) and nitrogen (RNS) species. This review assesses and discusses the current knowledge of NO signaling and its interaction with other signaling pathways, here focusing on the reported calcium (Ca^2+^) and hormonal signaling, under elevated GHGs along with the associated mechanisms underlying GHGs-induced stress in plants.

## Introduction

Nitric oxide (NO) was first described as nitrous air by Joseph Priestly in 1772 (Yu et al., 2014). However, NO production in plants was reported for the first time by Klepper (1979) about 43 years ago. Since then, our understanding of the diverse roles of NO in plant physiology and biology has increased significantly (Kolbert et al., 2019). The discovery of NO as a signaling molecule revealed novel facets of free radicals from their previous portrait as toxic by-products of oxidative metabolism to central regulators of diverse plant metabolic pathways. Unlike in animals where it is well established that a family of NO synthase (NOS) enzymes are the primary source of NO, the presence of such NOS enzyme in plants remains obscure and controversial. Whereas, some lines of evidence reported NOS-like activity in plants (del Rıo et al., 2004; Domingos et al., 2015; Phillips et al., 2018; Singh et al., 2021). During the last two decades, NO has gained momentum due to its multiple roles in plant growth and development (Sanz et al., 2015). As per some evidence (Arc et al., 2013; Signorelli & Considine, 2018), NO attenuates dormancy, while promoting seed germination, in crosstalk with the abscisic acid (ABA) signaling pathway. Similarly, several studies support that NO is a key player in the control of cell proliferation *via* a functional interaction with cytokinin (Shen et al., 2013). NO also plays an important role in the cell cycle (Correa-Aragunde et al., 2006; Novikova et al., 2017) and auxin-mediated activation of cell division (Ötvös et al., 2005). In the same way, Sánchez-Vicente et al. (2021) indicated that NO altered the pattern of auxin maxim and PIN-FORMED1 (regulates auxin basipetal transport) during shoot development. In addition, it is well established that NO is a key signaling molecule during abiotic or biotic stress conditions in plants (Magalhaes et al., 1999; Hancock & Neill, 2019; Singh et al., 2021). Available data suggest that NO generation in plants occurs by at least eight prominent processes that include enzymatic and nonenzymatic (Khan et al., 2014; Kolbert et al., 2019; Hussain et al., 2022).

Carbon dioxide (CO_2_), methane (CH_4_), nitrous oxide (N_2_O), and ozone (O_3_) play important roles in plant physiology. CO_2_ is required for photosynthesis and is fixed to store energy in the form of carbohydrates (Blankenship, 2021). Although the utilization of CO_2_ can be affected by factors such as light, water, nutrition, humidity and temperature, the atmospheric CO_2_ concentration has a greater influence. The increase in CO_2_ level has been shown to result in increased growth rate and biomass production (Ainsworth et al., 2004; Thompson et al., 2017). From another perspective, Sigurdsson (2001) reported variabilities in the response of plants to elevated CO_2_. Similarly, CH_4_, previously considered a physiological inert gas, is currently emerging as a signaling molecule that would interact with reactive oxygen (ROS) or nitrogen (RNS) species during abiotic or biotic stress events (Li et al., 2020; Wang et al., 2020). In the same way, N_2_O, of which the molecular mechanism underlying its production has been widely investigated, was reported to be produced in the mitochondria of plants from NO. In the soil, N_2_O is formed during the nitrification and denitrification processes (Lenhart et al., 2019; Timilsina et al., 2020a). Meanwhile, O_3_ causes both beneficial outcomes for plants and the environment (Pasqualini et al., 2009; Wargent & Jordan, 2013; Mukherjee, 2022; Yin et al., 2022), and oxidative stress, which may result in cell death (Riehl Koch et al., 1998; Rao & Davis, 2001). Although CO_2_, CH_4_, N_2_O, and O_3_ play beneficial roles in plant physiology and biology, these molecules have been identified as potent greenhouse gases (GHGs) (Khalil & Aslam, 2009; Timilsina et al., 2020b; Timilsina et al., 2022). However, in terms of global warming potential (GWP), N_2_O and CH_4_ come on top with a GWP of 300 times and 25 times, respectively, greater than that of CO_2_ in the atmosphere.

Studies show that high amounts of atmospheric CO_2_ (Niu et al., 2011), CH_4_ (Li et al., 2020), N_2_O, and O_3_ (Sharma & Davis, 1995) trigger various signaling cascades that serve as messengers to activate the adequate defense system to tackle the stress. During these events, plants enhance the production of ROS, such as hydrogen peroxide (H_2_O_2_), superoxide anion ( ${\text{O}}_{2}^{•-}$ ), hydroxyl radical (·OH^−^), singlet oxygen (^1^O_2_), and RNS (NO, peroxynitrite (ONOO^−^), etc.). In the process, NO signaling has proven essential and plays a central role. NO interacts with other signaling pathways, and the results of this interaction confer beneficial outcomes for plants. Generally, ROS and RNS are produced by plants under normal conditions and are harmless at low concentrations. However, upon stress induction by either abiotic stimuli or living organisms, the production of ROS and RNS increases up to the point of causing oxidative or nitro-oxidative stress, which may result in oxidative damage and culminate in cell death. To alleviate the detrimental effects of ROS or RNS overproduction, plants activate antioxidant (enzymatic and non-enzymatic) systems and induce several stress-responsive genes as part of the adaptive response mechanisms toward stress tolerance. This review assesses the current knowledge of the regulatory role of NO in plants under elevated CO_2_, CH_4_, N_2_O, or O_3_. This work also highlights the crosstalk between NO signaling and the above-mentioned potent GHGs, as well as the causative effects of elevated CO_2_, CH_4_, N_2_O, or O_3_ on NO production and signaling events. Likewise, we discuss the possible interplay between CO_2_, CH_4_, or N_2_O-induced stress with NO and other stress signaling pathways in plants to maintain a balanced reduction-oxidation status.

## Exogenous carbon dioxide induces nitric oxide in plants

In the current era identified as the Anthropocene, CO_2_ is the most important GHG (considering its emission abundance) emitted globally through human activities (Lashof and Ahuja, 1990; Butler and Montzka, 2016; Liu et al., 2020). Studies investigating NO signaling or biosynthesis in plants increased with climate change, of which the impact on the five critical dimensions of the sustainable development goals (SGDs, also known as the 5Ps: people, planet prosperity, peace and patnership) is no longer to be demonstrated. Like in the event of drought (Xiong et al., 2012; Lau et al., 2021), salinity (Bhardwaj et al., 2021; Fatima et al., 2021), heat stress (Kong et al., 2012; Parankusam et al., 2017), flooding (Khan et al., 2019; Da-Silva and do Amarante, 2022; Park et al., 2022), or heavy metal toxicity (Saxena and Shekhawat, 2013; Cerana and Malerba, 2015), elevated CO_2_ level triggers NO production and activates NO signaling in plants. Under these conditions, several stress-responsive pathways, including hormonal, Ca^2+^ (Besson-Bard et al., 2008; Niu et al., 2011) are induced, during which process NO plays a preponderant role. Niu et al. (2011) observed that elevated CO_2_ caused an increase in carbohydrates production, which in turn activated the auxin or ethylene-related signal transduction pathways that subsequently induced the production of endogenous NO.

## Elevated carbon dioxide induces nitric oxide-mediated nitrogen uptake and assimilation

Carbon dioxide supplementation enhances the root and shoot growth, resulting in the rapid growth of plants. This could be explained, in part, by the greater uptake of nutrients from soil mediated by the enhanced root development (Yue et al., 2009; Thompson et al., 2017). Under these conditions, available nutrients can be exhausted rapidly in the soil and plants may experience nutrient shortage or deficiency at their advanced growth stage. To sustain an increased growth rate under high CO_2_ conditions, plants will require higher amounts of inorganic nutrients, including nitrogen. To compensate the gap created due to nutrient deficiency, mineral fertilizers are applied (Wong, 1979; Tissue et al., 1997; Stitt and Krapp, 1999). However, excessive N-rich fertilizer applications cause an increase in CH_4_ and N_2_O production (Takeda et al., 2022; Timilsina et al., 2020a; Xu et al., 2016). Nitrogen is the most abundantly used essential macronutrient in agriculture. Nitrogen is available to the plant as nitrate (NO_3_) and NH_4_, with NO_3_ being the major form of nitrogen taken up by plants. The efficiency of nitrogen use by plants is mediated by several genes encoding nitrate reductase (NR) or belonging to five distinct high-, dual-, or low-affinity NO_3_ transporters protein families, including NRT1, NRT2, chloride channel (CLC), and slow anion channel-associated/slow anion channel-associated homologs (SLAC/SLAH) (Buchanan et al., 2015). In higher plants, NR is the first enzyme and the rate-limiting factor in the NO_3_ assimilation pathway (Buchanan et al., 2015). NR undergoes changes under elevated CO_2_ as reported by Stitt and Krapp (1999). As previously reported, elevated CO_2_ increases the use efficiency of organic nitrogen (NUE) (Wong, 1979; Hocking and Meyer, 1991a; Hocking and Meyer, 1991b; Pettersson et al., 1993; Rogers et al., 1993; McKee and Woodward, 1994). This could be partially explained by the low NO_3_ and NH_4_ contents (Purvis et al., 1974; Hocking and Meyer, 1985; Hocking and Meyer, 1991b; Yelle et al., 1987).

As per some evidence, the activity of NR varies across plant species, and different conditions under the influence of NO (Du et al., 2016; Adavi and Sathee, 2019). Thus, plants exposed to elevated CO_2_ recorded differential patterns, including an increase (Geiger et al., 1998; Constable et al., 2001; Hofmann et al., 2013; Du et al., 2016), a decrease (Stitt and Krapp, 1999; Matt et al., 2001), or unaffected (Agüera et al., 2006; Natali et al., 2009) NR activity. Interestingly, a study conducted by Frungillo et al. (2014) revealed that *S*-nitrosothiols regulate NO production and storage in plants through the nitrogen assimilation pathway. The authors emphasized that although NO production is mediated by various enzymatic and nonenzymatic pathways (including L-arginine), some amount of NO_2_ reduced from NO_3_ in the nitrogen metabolism is transported to the chloroplast. There, NO_2_ is converted to NH_4_ for further incorporation into amino acids, as part of the assimilation process. Another fraction of NO_2_ is converted to NO in the cytosol (Zheng et al., 2013).

In higher plants, *S-*nitrosoglutathione (GSNO, a stable, mobile, less toxic form of NO) is the major cellular reservoir of NO, and its accumulation is controlled by GSNO reductase (GSNOR1 that negatively regulates the process of protein *S*-nitrosation, thus controlling endogenous NO levels). GSNOR catalyzes the reduction (irreversible reaction) of GSNO to glutathione disulfide (GSSG, the oxidized glutathione form of GSH) and ammonia (NH_3_) (Yun et al., 2011; Medina-Rivera et al., 2015; Jahnová et al., 2019; Khajuria et al., 2019; Hurali et al., 2022). It was reported that GSNO exerts an inhibitory effect on NO_3_ uptake and reduction, which would occur *via* the inhibition of NR activity. Similarly, GSNOR catalyzes the reaction of NO with GSH (the reduced glutathione) (Feechan et al., 2005; Rustérucci et al., 2007). According to Chaki et al. (2009), the activity of GSNOR helps balance the cellular (RNS) reduction-oxidation (redox) homeostasis under various stressful conditions. Likewise, NO, which is one of the end products of the nitrogen metabolism, inhibits the activity of GSNOR. In turn, the latter prevents the degradation of GSNO. Therefore, building on the above mechanism, Frungillo et al. (2014) suggested that NO feedback regulates its flux *via* the assimilation of nitrogen by controlling its bioavailability and modulating its own consumption; knowing that high amounts of NO and its derived SNO result from the N assimilation pathway.

## Crosstalk between nitric oxide and salicylic acid signaling under elevated carbon dioxide

Plant hormones are key players in plant growth and development, operating either antagonistically or in synergy with one another. Plant hormones are also well-known for their involvement in signaling events during abiotic or biotic stress. For instance, salicylic acid (SA) signaling regulates plant response to stress (Khan et al., 2015). Other studies demonstrated that the interplay between NO and SA signaling pathways activates appropriate defense mechanisms and enhances resistance to a wide range of plant pathogens (Klessig et al., 2000). According to Li et al. (2019), SA acts upstream of NO under elevated CO_2_, which in turn mediates the induction of flavonoid biosynthesis in tea (*Comellia sinensis* L.). The authors observed that SA, NO, and flavonoid contents increased in plants exposed to elevated CO_2_.

## Elevated carbon dioxide-induced nitric oxide generation regulates stomatal conductance

Stomata are epidermal pores through which gas exchange is regulated, including CO_2_ assimilation. Several studies support that the regulation of stomatal aperture size is achieved through a complex sensory and signaling network (Kim et al., 2010; Merilo et al., 2014). Stomata aperture facilitates more CO_2_ uptake, which enhances photosynthesis (a process by which CO_2_ is captured from the atmosphere and is converted to sugar as a source of energy for plant cell, growth and development, as well as plant fitness). It is widely known that plant guard cells are essential for photosynthesis and transpiration, and the stomata aperture is sensitive to environmental stimuli. Recent progress in stress signaling revealed that high levels of CO_2_-induced stomata closure suggest ROS such as hydrogen peroxide (H_2_O_2_), as key factors. ROS are major regulators of stomatal conductance in response to internal or external stress inducers (Sun et al., 2021). Pharmacological and genetic studies showed that NADPH oxidases and cell wall peroxidases-mediated ROS regeneration participate in elevated CO_2_-induced stomatal closure. Whereas, elevated CO_2_-mediated inhibition of light-induced stomatal opening would rely on ROS derived from NADPH oxidases, and not from cell wall peroxidases (He et al., 2020). From another perspective, Wang et al. (2015) demonstrated that NR and NOS-like enzymes are involved in CO_2_-induced NO accumulation in plants, under which conditions the regulation of stomata and photosynthesis are differentially affected. In a converse approach, Cornic (2000) observed the inhibition of photosynthesis by a decrease in stomatal aperture, as a result of a decrease in CO_2_ acquisition. In the same way, Garcia-Mata and Lamattina (2007) reported that light-induced stomatal opening *via* calcium (Ca^2+^) and NO-mediated signaling pathways is inhibited by ABA. The authors suggested that NO and Ca^2+^ are active components of ABA-induced stomatal closure. Similarly, Chater et al. (2015) suggested that ABA synthesis and signaling are required during elevated CO_2_-induced stomatal regulation.

**Figure 1:**
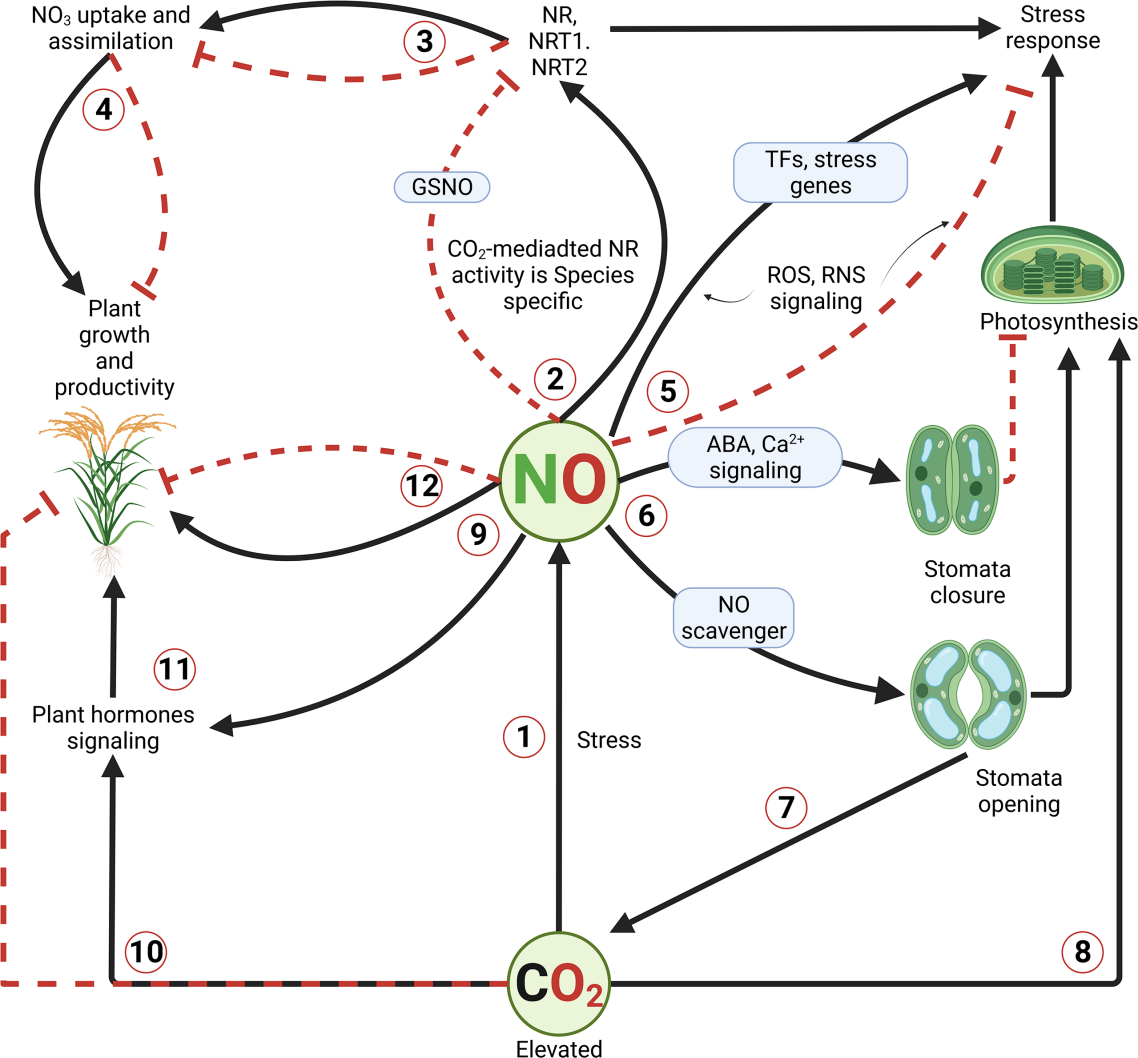
Illustration of elevated carbon dioxide (CO_2_)-mediated NO signaling in plants. Unlike other abiotic stresses, elevated CO_2_ levels have been shown to have both beneficial and detrimental effects on plant growth and development. Beneficial effects are resumed in enhancing the photosynthetic capacity and promotion of growth and development. However, at a certain accumulation level, CO_2_ induces various signaling cascades, in which NO is said to play a central role. In the process, nitrate reductase and transporter encoding genes are induced, along with the Ca^2+^ and ABA signaling, ROS and/or RNS signaling, resulting in differential stomatal conductance, enhancement of NO_3_ uptake and assimilation, and eventually better growth and productivity. Continuous black lines with an arrow indicate positive regulation or induction, while dash red lines with a perpendicular bar denote negative regulation or inhibition in case of over production/accumulation of compounds.

Moreover, Hsu et al. (2018) supported that elevated CO_2_-mediated stomatal closure *via* an ABA-independent pathway would involve the OST1/SnRK2 (OPEN STOMATA 1/SNF1-related protein kinases 2) kinases family. In addition, studies have suggested that the signaling pathway for elevated CO_2_-induced stomatal closure shares several elements with but does not overlap with (Hu et al., 2010; Tian et al., 2015), the ABA signaling pathway in guard cells. Such induction of OST1 and its target SLOW-TYPE ANION CHANNEL 1 (SLAC1, also known as one of the five major NO_3_ transporter protein families), ROS and NO production, enhanced Ca^2+^ level (Webb et al., 1996; Xue et al., 2011; Shi et al., 2015; Geng et al., 2017). Lines of evidence demonstrated that NO acts as an important secondary messenger in guard cells during stomatal closure (Gayatri et al., 2013). As for Sami et al. (2021), NO enhances the photosynthetic efficiency, among other plant metabolisms. This evidence would be attributed to the increase in biomass, and greater leaf area, causing an increase in productivity (Lawlor and Mitchell, 1991). As for Haworth et al. (2016), elevated CO_2_ would cause a reduction in photosynthetic physiology in plants. A study conducted by De Souza et al. (2008) supported that elevated CO_2_ levels (720 ppm) increased photosynthesis in sugarcane plants compared to those grown under ambient (370 ppm) CO_2_ level. This evidence was supported in several CO_2_-enrichment studies (Lawlor and Mitchell, 1991). As illustrated in **Figure 1**, we have proposed a signaling model involving NO and other signaling pathways in plants exposed to high concentrations of CO_2_.

In plants, the cytoskeleton (a structure made up of filamentous proteins responsible for the morphology and intracellular organization of the cell) provides mechanical support to the cell and enables the cell to execute essential functions (Kost and Chua, 2002; Hawkins et al., 2013). The actin cytoskeleton plays a fundamental role in diverse biological processes in plants, such as cell division and expansion, organelle movement, vesicle trafficking, and the establishment of polar cell growth (Kost and Chua, 2002; Paez-Garcia et al., 2018). Drøbak et al. (2004) supported that, in addition to its role in maintaining cell shape and structure, the actin cytoskeleton and its associated elements serve as a key target in various signaling events, as well as a signal transducer. Furthermore, the actin cytoskeleton has been identified as a major target and an effector of various signaling cascades in plants, including Ca^2+^, mitogen-activated protein kinase/kinase (MAPK/MAPKK) signaling, phytohormone signaling, etc. (Hussey et al., 2006; Wang et al., 2011). Evident findings revealed that the reorganization of cytoskeleton components under stress conditions is regarded as a crucial cell survival response (Wang and Hussey, 2015; Soda et al., 2016a; Sampaio et al., 2022). As indicated in previous paragraphs, high CO_2_ levels-mediated changes in the stomatal conductance require ABA and ABA signaling (Chater et al., 2015). Stomatal movement is regarded as a means used by plants to increase their adaptability to environmental change. A recent review report discussing the progress on the dynamics of actin filaments and microtubules in the guard cell (Li et al., 2022) revealed that the cytoskeleton is an important factor influencing stomatal conductance. This implication would occur through the change in turgor pressure guard cells. Studies highlight that the shape, structure, and mechanics of guard cells are affected by cytoskeleton-mediated cell division and cell wall synthesis (Panteris et al., 2018; Woolfenden et al., 2018; Muroyama et al., 2020).

Advances in the molecular mechanism underlying NO signaling in plants revealed that NO regulates the function of target proteins through post-translational modifications (PTMs), including tyrosine nitration or *S*-nitrosation previously referred to as *S*-nitrosylation (Yemets et al., 2011), thus affecting their activity. Likewise, evidence support that the cytoskeleton is involved in the NO-signaling network (directly or indirectly); while, NO-mediated regulation of cytoskeleton functions occurs through PTMs. Meanwhile, Kasprowicz et al. (2009) found that the organization of the actin cytoskeleton is modulated *via* NO levels in a cell-type-specific fashion. Findings from diverse studies sustain the existence of a tight association between changes in atmospheric CO_2_ levels and structural adaptation in plants. Although studies present diverging views on the actual effects of high CO_2_ levels on photosynthesis, evidence revealed the alteration of plant structure by impairing the rate of cell division, cell expansion, and cell cycling due to high CO_2_ concentrations. Under these circumstances, metabolic changes could be induced at the cellular level (Masle, 2000; Sharma et al., 2014). Given the role of the cytoskeleton in the stress signaling events as portrayed earlier, coupled with the interplay between NO signaling and the cytoskeleton function in plants, it has become evident that the cytoskeleton plays a crucial role in the adaptive response against abiotic stimuli (Soda et al., 2016b), including elevated CO_2_.

## Interplay between nitric oxide and calcium signaling during elevated carbon dioxide

Depicting the mechanisms underlying plants response to abiotic stress helps improve the understanding of genetic factors associated with stress tolerance in plants. Although investigating the role of each signaling molecule independently provides some useful information on their level of implication in the regulatory mechanisms during abiotic stress events, a genome-wide approach gives new insights on the possible interactions between signaling molecules and their impact at the whole plant level. Generally, signaling pathways do not operate solo. Rather, they are activated along with other signaling networks with which they may establish a certain level of interaction. This interaction may occur in a balanced way since it is a high-energy demanding process consisting of antagonistic or synergetic relationships. This may result in what could be referred to as signaling cascades, allowing the induction or suppression of downstream adaptive response mechanisms. Just like other stress-related conditions where NO plays an active role (Ma et al., 2012; Niu et al., 2017; Sun et al., 2017; Khan et al., 2020), a study conducted by Wang et al. (2013a) revealed crosstalk between NO and Ca^2+^ signaling (also known as a secondary messenger) during episodes of elevated CO_2_, which led to enhanced lateral root development. The authors equally indicated that a CO_2_-mediated increase in NO production triggers the accumulation of cytosolic Ca^2+^ that acts as a co-factor for NO. In a converse approach, Young et al. (2006) investigated the CO_2_ signaling in guard cells and indicated that low–high CO_2_ transitions modulate the cytosolic Ca^2+^ transient patterns.

## Enhanced methane concentration triggers nitric oxide and ROS signaling in plants

**Figure 2:**
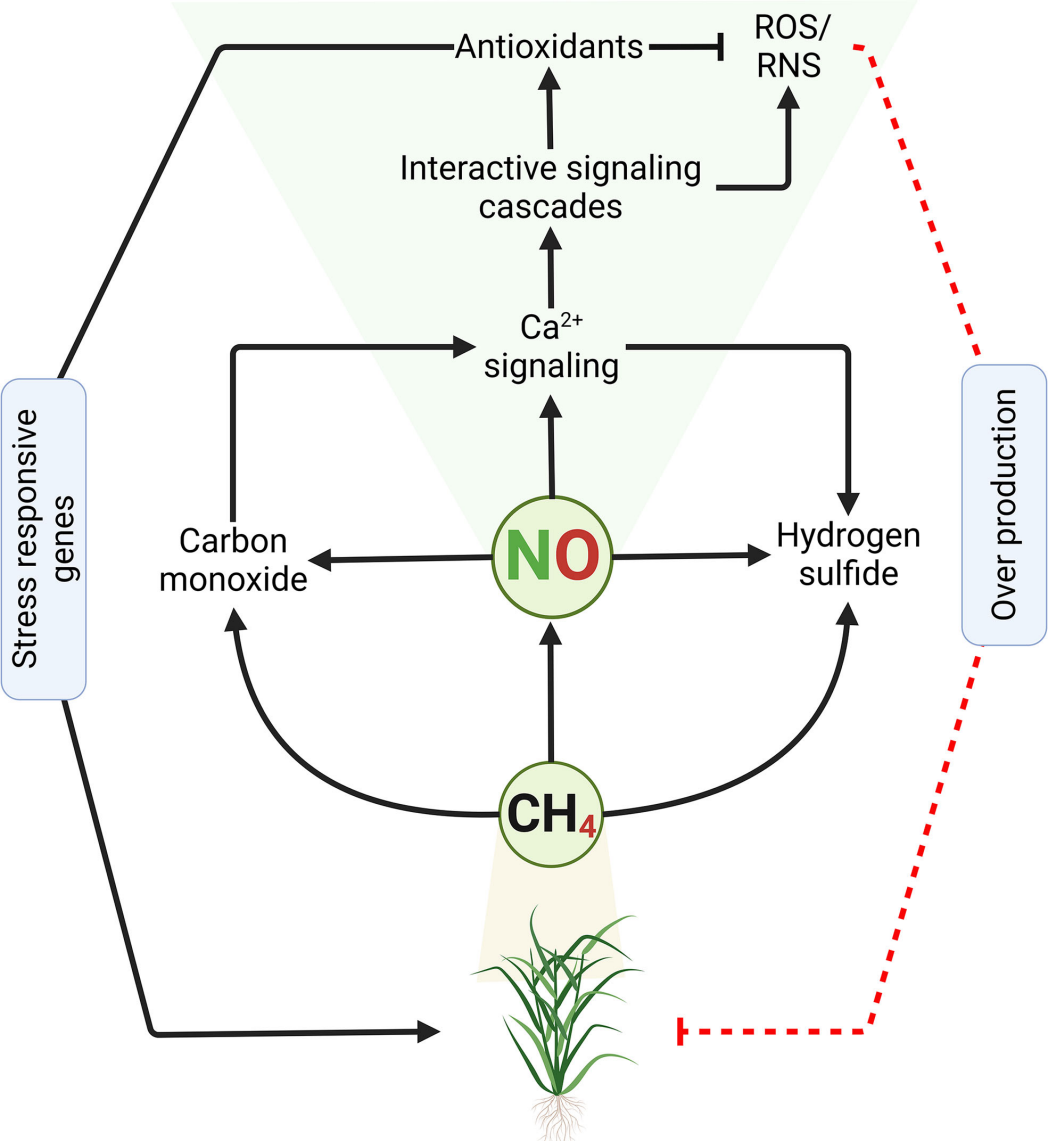
Methane induces nitric oxide accumulation in plants. High concentration of methane in the atmosphere or within the plant triggers activation of various signaling and biosynthetic pathways, including NO. NO requires Ca^2+^, among others, as cofactors to govern the adaptive response mechanisms towards abiotic stress in plants. The crosstalk between NO and Ca^2+^ along with other signaling cascades helps the plant to activate the appropriate defense system to tackle the stress, by inducing related stress-responsive genes and accumulation of various antioxidant systems that detoxify the effect of reactive oxygen (ROS) and nitrogen (RNS) species over accumulation. Persistent over production of ROS and/or RNS leads to oxidative stress/damage that may culminate to cell death and plant necrosis. Continuous black lines with an arrow indicate positive regulation or induction, while dash red line with a perpendicular bar denote negative regulation or inhibition.

Methane (CH_4_) was previously regarded as a physiologically inert gas but is now emerging as a possible signaling molecule in plants (Li et al., 2020). Some lines of evidence suggest that there could be an interaction between CH_4_ and ROS, as well as other signaling molecules such as NO, GSH, and hydrogen sulfide (H_2_S) (**Figure 2**). Li et al. (2020) indicated that CH_4_ production occurs through abiotic or biotic pathways. The latter is proposed to be the major pathway of CH_4_ production through the decomposition of organic compounds as well as microbial activity (Emmanuel and Ague, 2007; Fiebig et al., 2009; Wang et al., 2013b). CH_4_ exerts a protective effect through the reduction of oxidative stress (Wang, 2014). Similarly, Hu et al. (2018) observed that CH_4_ delayed post-harvest senescence by re-establishing redox homeostasis. Likewise, Wang et al. (2009) indicated that physically injured plants- and hypoxic conditions-mediated generation of ROS stimulated CH_4_ emission. These facts nourish the idea that the production of CH_4_ by plants would be part of a survival strategy during stress conditions. Zhang et al. (2018) exposed Mung bean plants to polyethylene glycol-induced osmotic stress and discovered that NO contributed to CH_4_-induced osmotic stress tolerance. In a converse approach, Qi et al. (2017) investigated the role of CH_4_ in inducing the development of adventitious roots in cucumber found that CH_4_ triggers accumulation NO. The interaction between NO and CH_4_ has recently been reported (Boros and Keppler, 2019). From another perspective, Zhang et al. (2018) reported that exogenous CH_4_ triggered NO production under polyethylene glycol-induced osmotic stress, and alleviated the inhibition of seed germination. Therefore, this evidence suggests that CH_4_-induced abiotic stress tolerance would be NO-dependent, which might involve NR and NOS-like protein.

Studies aiming at elucidating the unexplored facets of NO in plants discuss the possible interplay between NO and oxygen (O_2_) availability in plants. It is believed that NH_4_ and NO_3_ are the major sources of nitrogen for plants, with NO_3_ being the most abundant. O_2_ differentially influences GHGs emission patterns, acting on the activity of specific soil microorganisms, such as methanogens and methanotrophs. In addition, as indicated in previous paragraphs, the influential role of NO on nitrogen assimilation events positions this molecule at the core center of interest in various stress-related studies. It was reported that NO contributes to CH_4_-mediated induction of osmotic stress tolerance in mung bean (Zhang et al., 2018). Cucumber adventitious rooting was reported to be induced by CH_4_-rich water *via* heme oxygenase/carbon monoxide and Ca^2+^ pathways.

## Nitric oxide and nitrous oxide are tightly related

Nitrous oxide (N_2_O) is a potent GHG with a GWP much higher than CO_2_ and CH_4_. N_2_O is emitted by diffusion from soil or *via* plant transpiration (Chang et al., 1998). The release of N_2_O to the atmosphere by plants occurs during the nitrification and denitrification processes. The latter are mediated by soil microbial activities in the soil as well as in the shoot of plants *via* the action of certain enzymes of the nitrogen metabolism, in which NO takes an active part (Timilsina et al., 2020a). It is established that NO_3_ is a precursor for N_2_O formation in both plants (Hakata et al., 2003; Lenhart et al., 2019) and soil (Andrew et al., 2012). In their study, Burlacot et al. (2020) found that the conversion of NO to N_2_O in *Chlamydomonas reinhardtii (*Alga) occurs during the photosystem I (PSI)-dependent photoreduction of the photosynthesis. This was similarly observed by Schützenmeister et al. (2020). Meanwhile, Gupta et al. (2005) observed that in higher plants, only root mitochondria, but not leaf mitochondria reduce nitrite to NO. Both N_2_O and NO are known as NOx, and react with volatile organic compounds and hydroxyl, resulting in organic NO_3_ and nitric acid (HNO_3_) formation (Cassia et al., 2018). Unlike in animal and prokaryotic organisms (Arai et al., 2003), the molecular mechanism underlying N_2_O-induced stress in plants has not been elucidated. Nevertheless, considering the NO-dependent N_2_O formation, an imbalance in NO synthesis or signaling would affect N_2_O production in plants.

## Ozone-induced stress triggers nitric oxide signaling in plants

Like during other abiotic stresses, when plants are exposed to ozone (O_3_), they undergo various physiological and biochemical changes. In the process, various signaling pathways are activated and act as messengers to trigger an adequate adaptive response mechanism towards stress tolerance, along with the induction or suppression of stress-related genes (Delaney et al., 1994; Sharma and Davis, 1995; Pasqualini et al., 2009). Under the same conditions, antioxidant systems are activated (Nogués et al., 2008; Caregnato et al., 2010), including GSH and ascorbate-GSH cycle (Zhang et al., 2020). The molecular basis of O_3_-induced stress signals in plants has been widely investigated, of which an SA-dependent signaling pathway was reported to be activated in plants exposed to O_3_ (Sharma and Davis, 1997). As indicated in previous paragraphs, GSNOR catalyzes the reaction of NO and GSH. A study conducted by Alscher and Hess (1993) sustained that O_3_ treatment affects the glutathione metabolism in plants. Similarly, Gupta et al. (1991) observed an overtime increase in GSH, GSSG, and total GSH levels in poplar upon O_3_ treatment. As to Guri (1983), a correlation exists between differential O_3_ sensitivity and accumulation levels of GSH and the activity GR in tolerant *Phaseolus vulgaris* cultivars.

Furthermore, a study revealed that dual application of NO and O_3_ induced a large set of stress-responsive genes, therefore suggesting their possible interplay (Ahlfors et al., 2009a). In the same way, Ahlfors et al. (2009) demonstrated that O_3_-mediated NO accumulation coincided with the hypersensitive response (HR) in *Arabidopsis*, followed by O_3_-mediated induction of SA biosynthesis and signaling pathway genes, and ethylene accumulation. Interestingly, Xu et al. (2012) supported that NO_3_ reductase is responsible for O_3_-triggered NO generation and secondary metabolites in *Ginkgo biloba* plants. Moreover, useful information that would allow enhancing our understanding of the molecular mechanism underlying O_3_-mediating induction of various signaling pathways and ROS in plants is well summarized by Hasan et al. (2021), who highlighted the role of key protein families associated with the nitrogen metabolism, such as slow anion channel 1 (SLAC1) and that of diverse phytohormone signaling pathways, which regulate stomatal conductance. Likewise, Domingos et al. (2015) highlighted the multidimensional roles of NO in gas signaling in plants. In a recent study, Mukherjee (2022) emphasized the forefront role of NO in mediating O_3_-induced stress signaling in plants.

Moreover, land plants rely on light for photosynthesis, and leaves serve as light-capturing organs. However, as an undesired corollary, they also absorb damaging light, especially ultraviolet B (UV-B) radiation (280–320 nm waveband of the solar irradiation) (Gupta et al., 2011), a kind of UV light that directly affects plants and microorganisms, and alters the species-specific interactions (Vanhaelewyn et al., 2020), and causes cell death in plants (Nawkar et al., 2013), which was manifested by cell shrinkage, condensation of chromatin in perinuclear areas, and formation of micronuclei in UV-B treated BY-2 tobacco cells (Lytvyn et al., 2010). On the one hand, exposure to UV-B radiation has been shown to impair the genome stability of plants by damaging nucleic acids (Ries et al., 2000; Tanaka et al., 2002). The good news is that the O_3_ layer in the stratosphere helps absorb UV-B radiation. However, in modern times, exposure to UV-B radiation increases gradually due to the thinning of the protective O_3_ layer caused by human activities (Andrady et al., 2010). Plants have evolved sophisticated strategies to adapt to the incidence of UV-B light, such as increasing leaf thickness, UV-B reflective properties, and the cellular levels of UV-B absorbing metabolites. In the UV-B signal transduction network, members of the bZIP (basic leucine zipper) transcription factor (TF) family, ELONGATED HYPOCOTYL5 (HY5, a key effector of the UV RESISTANCE LOCUS 8 (UVR8)) and HY5-HOMOLOG (HYH) that mediate several photomorphogenic pathways, and the E3-ubiquitin ligases (COP1) are required for UV-B-induced gene expression (Oravecz et al., 2006; Tohge et al., 2011). Reports indicate that low doses of UV-B induce distinct signaling pathways from the high doses-stress response pathways (Frohnmeyer et al., 1999; Jenkins et al., 2001; Brown et al., 2005). In addition to UV-B radiation, Danon and Gallois (1998) investigated the molecular response of *Arabidopsis thaliana* plants exposed to UV-C radiation (10–50 kJ/m^2^), and observed the induction of an oligonucleosomal DNA fragmentation, which characterizes apoptotic-like changes in the nucleus, similar to that observed in human cells (Martin & Cotter, 1991). Reports have shown that UV-C (below 280 nm) is not physiologically relevant to plants because it is effectively intercepted by the earth’s stratosphere (Brash, 1997); however, UV-C radiation yields similar DNA photoproducts to that obtained with UV-B radiation, which reaches the surface of the earth. Thus, several studies employed UV-C radiation to explorer potential DNA damages. In this context, Danon et al. (2004) observed that overexposure of *Arabidopsis* plants to UV-C radiation induced programmed cell death (PCD), the latter being suggested to be mediated by Caspase-like activities, which in turn would modulate DNA fragmentation. In the process, Caspase inhibitors suppress DNA fragmentation and cell death, where two *AtDAD1* and *AtDAD2*, earlier identified as homologs of *Defender against Apoptotic Death-1*, are proposed to suppress the onset of DNA fragmentation while supporting an involvement of the endoplasmic reticulum in this form of the plant PCD pathway.

On the other hand, UV radiation triggers the production of free radicals, including ROS and NO (Tossi et al., 2009; Zhang et al., 2011), as well as the counteracting plant defense antioxidants such as ascorbate and glutathione (Jansen et al., 1998; Santa-Cruz et al., 2014). The involvement of NO signaling in mediating plant response to UV-B-induced oxidative stress has been proposed. In this regard, Lytvyn et al. (2016) highlighted the multiple functions of inositol biosynthesis in plants exposed to UV-B. Their study revealed that the response mechanism to NO-dependent oxidative stress induction in *Arabidopsis* involves the inositol-3-phosphate synthase (IPS1), a key enzyme for biosynthesis of *myo*-inositol and its derivatives. Other studies showed that the role of ROS are important elements of a wide signaling web that composes with other signaling mediators to activate cellular protective mechanisms in response to UV-B radiation. For instance, *Arabidopsis* plants treated with the NO scavenger PTIO (2-phenyl-4,4,5,5-tetramethylimidazolin-L-oxyl-3-oxide) and/or with L-NAME (N^G^-monomethyl-L-arginine), an NOS (NO synthase) inhibitor, suppressed the induction of chalcone synthase (CHS)-encoding gene; therefore suggesting that UV-B-triggered the expression of *CHS* would require NO (Soheila et al., 2001). One should keep in mind that the presence of NOS in planta remains obscure. In another perspective, Tossi et al. (2014) showed that the *Arabidopsis* UVR8 photoreceptor regulates plants’ response to UV-B-induced stomatal closure in a NO-dependent manner. Likewise, Wu et al. (2016) revealed that the induction of anthocyanin under UV-B radiation is regulated by the interaction between H_2_O_2_, NO and UVR8 in radish. Similarly, Cassia et al. (2019) proposed that UV-B triggers the accumulation of ABA, which increases the production of H_2_O_2_ and NO. In the process, the UV-B receptor UVR8 is activated. The latter is stabilized by endogenous NO followed by the induction of HY5 TF. In turn, HY5 TF has the potential to regulate the expression and activity of NR, as well as CHs and chalcone isomerase (CHI) resulting in a downstream increase in flavonoid and anthocyanin contents capable of absorbing UV-B radiation and scavenging ROS.

## Conclusion and perspectives

Nitric oxide (NO) is at the core center of interest in many biosciences and environmental-related research programs, mainly due to its involvement in almost all physiological and biological processes in plants. Since the initial report of NO in plants about four decades ago, our understanding of the molecular mechanism underlying NO biosynthesis and signaling in plants increased during the last two decades, and the physiology of NO has been widely investigated under both normal and stressful conditions. In this work, we assessed and presented the current knowledge on the regulatory network involving NO and its derived molecules in the adaptive response mechanism of plants towards elevated CO_2_, CH_4_, N_2_O, O_3_ or UV light. This review also gives insights into the interaction of NO with other signaling pathways and highlights the involvement of the nitrogen metabolism and the flavonoid pathway genes in NO-mediated stress signaling during elevated GHGs. These GHGs present both beneficial outcomes and detrimental effects to the plant, depending on their level of accumulation in the atmosphere. Therefore, taking advantage of the current understanding of NO in its diverse dynamic roles in various stressful conditions, NO can be regarded as a game changer in the efforts towards the mitigation of the impact of climate change, while providing a novel path to enhancing the resilience of agricultural and food production systems. In the context of climate change, depicting the molecular basis of NO-mediated plants’ response to elevated CO_2_, CH_4_, N_2_O, or O_3_ would help elucidate the regulatory mechanisms underlying plants’ response to these GHGs. In addition, exploring the interplay between NO-mediated nutrient acquisition and use efficiency, and the associated defense mechanisms against GHGs would provide more insights towards a paradigm shift for a more resilient agriculture.

## Author contributions

NK and B-GM designed the work, conducted the literature mining, and drafted the paper, S-ML, YK, DL, and G-ML contributed to the literature review and manuscript preparation. B-WY and J-HL conceived, revised and edited the manuscript and supervised the project. All authors contributed to the article and approved the submitted version.

## Funding

This work was supported by the Cooperative Research Program for Agriculture, Science and Technology Development (Project No. PJ01707301) and “2019KoRAA Long-Term Training Program” of the Rural Development Administration, Korea, and the Basic Science Research Program through the National Research Foundation of Korea (NRF) funded by the Ministry of Education (Grant No.2020R1I1A3073247).

## Conflict of interest

The authors declare that the research was conducted in the absence of any commercial or financial relationships that could be construed as a potential conflict of interest.

## Publisher’s note

All claims expressed in this article are solely those of the authors and do not necessarily represent those of their affiliated organizations, or those of the publisher, the editors and the reviewers. Any product that may be evaluated in this article, or claim that may be made by its manufacturer, is not guaranteed or endorsed by the publisher.
